# A S100A14-CCL2/CXCL5 signaling axis drives breast cancer metastasis

**DOI:** 10.7150/thno.42087

**Published:** 2020-04-27

**Authors:** Xukun Li, Minjie Wang, Tongyang Gong, Xuemeng Lei, Ting Hu, Maoqing Tian, Fang Ding, Fei Ma, Hongyan Chen, Zhihua Liu

**Affiliations:** 1 The State Key Laboratory of Molecular Oncology, National Cancer Center/National Clinical Research Center for Cancer/Cancer Hospital, Chinese Academy of Medical Sciences and Peking Union Medical College, Beijing, 100021, China; 2 Department of Clinical Laboratory, National Cancer Center/National Clinical Research Center for Cancer/Cancer Hospital, Chinese Academy of Medical Sciences and Peking Union Medical College, Beijing, 100021, China; 3 Medical College, Guizhou University, Guizhou, 550025, China; 4 Department of Medical Oncology, National Cancer Center/National Clinical Research Center for Cancer/Cancer Hospital, Chinese Academy of Medical Sciences and Peking Union Medical College, Beijing, 100021, China

**Keywords:** Breast cancer, Metastasis, S100A14, CCL2, CXCL5

## Abstract

**Rationale**: Chemokines contribute to cancer metastasis and have long been regarded as attractive therapeutic targets for cancer. However, controversy exists about whether neutralizing chemokines by antibodies promotes or inhibits tumor metastasis, suggesting that the approach to directly target chemokines needs to be scrutinized.

**Methods**: Transwell assay, mouse metastasis experiments and survival analysis were performed to determine the functional role of S100A14 in breast cancer. RNA-Seq, secreted proteomics, ChIP, Western blot, ELISA, transwell assay and neutralizing antibody experiments were employed to investigate the underlying mechanism of S100A14 in breast cancer metastasis. Immunohistochemistry and ELISA were performed to examine the expression and serum levels of S100A14, CCL2 and CXCL5, respectively.

**Results**: Overexpression of S100A14 significantly enhanced migration, invasion and metastasis of breast cancer cells. In contrast, knockout of S100A14 exhibited the opposite effects. Mechanistic studies demonstrated that S100A14 promotes breast cancer metastasis by upregulating the expression and secretion of CCL2 and CXCL5 via NF-κB mediated transcription. The clinical sample analyses showed that S100A14 expression is strongly associated with CCL2/CXCL5 expression and high expression of these three proteins is correlated with worse clinical outcomes. Notably, the serum levels of S100A14, CCL2/CXCL5 have significant diagnostic value for discerning breast cancer patients from healthy individuals.

**Conclusions**: S100A14 is significantly upregulated in breast cancer, it can promote breast cancer metastasis by increasing the expression and secretion of CCL2/CXCL5 via RAGE-NF-κB pathway. And S100A14 has the potential to serve as a serological marker for diagnosis of breast cancer. Collectively, we identify S100A14 as an upstream regulator of CCL2/CXCL5 signaling and a metastatic driver of breast cancer.

## Introduction

Metastasis is a major cause of death for cancer patients. The process of metastasis is composed of a cascade of linked sequential steps, including tumor invasion, migration, host immune escape, extravasation, angiogenesis and tumor growth 1, 2. It is well established that the tumor microenvironment (TME) is an important determinant of tumor behavior in cancer development and metastasis. Tumor-associated macrophages (TAMs), the most abundant immune-related stromal cells in the TME, promote tumor invasion and metastasis. The alternatively activated TAM phenotype is regulated by specific tumor-derived chemokines that are important components of cancer-related inflammation 3, 4. Chemokines have long been associated with cancers, where they play key roles in orchestrating the recruitment and positioning of leukocytes 5. Among numerous chemokines, CCL2 and CXCL5 are of particular interest as their expression is elevated in diverse tumor types (e.g. breast cancer) and correlates with poor prognosis of patients 6, 7. CCL2, a key chemokine released by macrophages, functions through binding to CCR2 and is a major determinant of macrophage recruitment, positioning and polarization in tumors 8, 9. CXCL5, a member of the CXC-type chemokine family that directs chemoattractant and activation effects on neutrophils via binding to the CXCR2 receptor, is also a potent macrophage chemoattractant 10-12. Although chemokines have long been regarded as attractive therapeutic targets for cancer, controversy exists about whether neutralizing chemokines by antibodies promotes or inhibits tumor growth and metastasis 13. Therefore, there is an urgent need to uncover molecular mechanisms for chemokine signaling in cancers to enable better chemokine targeted therapies.

The S100 family proteins possess a wide range of intracellular and extracellular functions and have been implicated in tumorigenesis and tumor progression. Interestingly, S100 proteins have been strongly connected to the communication between cancer cells and stromal cells, thereby contributing to tumor metastasis 14, 15. For example, the S100A7/8/9-IRAK1 signaling pathway has been identified as an important driver of breast cancer progression 16. In the case of S100A14, our previous studies found that S100A14 promotes cancer cell migration and invasion by MMP2 through p53-dependent transcriptional repression 17, it is unknown whether and how S100A14 promotes breast cancer progression.

Here, we showed that S100A14 promotes breast cancer metastasis. By analyzing S100A14-regulated transcriptome and proteome in breast cancer cells, we found that S100A14 regulates the expression of a panel of inflammatory chemokines and cytokines. Mechanistic studies showed that S100A14 facilitates the translocation of NF-κB to the nucleus to directly activate the transcription of CCL2 and CXCL5. The relevance and significance of the S100A14-CCL2/CXCL5 axis was validated by analysis of patient data from our own samples and public database. Collectively, our results suggest that targeting S100A14 may inhibit aberrant CCL2/CXCL5 signaling in metastatic breast cancer.

## Materials and Methods

### Cell culture

MCF7, MCF10A, T47D, SKBR3, BT549, MDA-MB-231 and HEK293T cells were purchased from the American Type Culture Collection (ATCC, Manassas, VA, USA). MCF10AT, MCFCA1h, MCFCA1a and 4T1 cells were provided by professor Guohong Hu (Shanghai Institutes for Biological Sciences, China). Phoenix cells were provided by professor Han You (Xiamen University, China). MCF7, T47D, SKBR3, BT549, MDA-MB-231 and HEK293T cells were cultured according to the manufacturer's recommendations. MCF10A, MCF10AT, MCFCA1h and MCFCA1a were maintained in Dulbecco's modified Eagle's medium: nutrient mixture F-12 (DMEM/F-12) supplemented with 5% equine serum, 2 mM L-glutamine, 20 ng/mL EGF, 0.01 mg/mL insulin, 500 ng/mL hydrocortisone, 100 ng/mL cholera toxin, 100 units/mL streptomycin and 100 units/mL penicillin. 4T1 cells were maintained in DMEM supplemented with 10% fetal bovine serum, 1× NEAA, 100 units/mL streptomycin and 100 units/mL penicillin. Phoenix cells were maintained in DMEM supplemented with 10% fetal bovine serum, 100 units/mL streptomycin and 100 units/mL penicillin. All of the cells were authenticated by short tandem repeat (STR) analysis and regularly tested for mycoplasma contamination.

### Plasmids, lentiviruses, retroviruses, and transduction

The full-length cDNA of human S100A14 and mouse S100A14 was cloned into the pLVX-IRES-Neo and pMSCV-puro vector, respectively. Two sgRNA oligos targeting S100A14 were cloned into the LentiCRISPR v2 vector. For lentivirus packaging, 6.5 μg pCMV Δ8.9, 3.5 μg VSVG, 2.5 μg PLP2 together with 7.5 μg pLVX-IRES-Neo/pLVX-IRES-Neo-hS100A14 or 7.5 μg LentiCRISPR v2/LentiCRISPR v2-sgRNA was cotransfected into HEK293T cells with Lipofectamine 2000. For retrovirus packaging, 24 μg pMSCV-puro/pMSCV-puro-msS100A14 was transfected into Phoenix cells with Lipofectamine 2000. After 48 h, the supernatant was harvested, centrifuged at 3,000 g for 20 min at 4 °C, and then filtered with a 0.45 μm syringe filter. For viral transductions, 2×10^5^ cells/well were seeded in 6-well culture plates and infected with viruses plus polybrene (8 μg/mL) for 48 h. To obtain stable cell lines, cells were selected for one-to-two weeks with 550 μg/mL geneticin or 1 μg/mL puromycin.

### RNA isolation and quantitative real-time PCR (qRT-PCR)

Total RNA was extracted with TRIzol Reagent (Life Technologies). Complementary DNA was synthesized by a QuantScript RT kit (KR103, TIANGEN) according to the manufacturer's instructions. Quantitative real-time PCR was carried out using PowerUp^TM^ SYBR^TM^ Green Master Mix (A25742, Applied Biosystems). Data were analyzed via the comparative Ct method. β-actin was employed as an internal control. The primers are listed in **Table S1**.

### siRNA transfection

siRNAs targeting NF-κB gene and siRNA control were purchased from GE Dharmacon. Cells were transfected with 60 nM of siRNA using Lipofectamine 2000 (Invitrogen) according to the manufacturer's instructions. Sequences of siRNAs are available in **Table S2**.

### Western blot

The protein was extracted with RIPA lysis buffer containing cocktail and quantified with a BCA kit (23225, Thermo Fisher Scientific). Protein extracts were separated by 10% Next Gel (1B1724, Amresco), then transferred to a PVDF membrane (Immobilon-P, Millipore). The membrane was blocked with 5% skim milk for 2 h at room temperature, incubated with a primary antibody overnight at 4 °C and a secondary antibody for 2 h at room temperature. The membrane was washed four times with TBST for 10 min each time and finally detected with the LAS4000 mini system (GE Healthcare, Piscataway, NJ, USA). The antibodies used are listed in **Table S3**.

### Cell proliferation assay

Cells were seeded in 96-well culture plates (2.5×10^3^ /100 μL) and cultured in an incubator. At each indicated time point, 100 μL of fresh medium containing 10 μL of CCK-8 (CK04, DOJINDO) was added to each well. After incubation for 1 h at 37 °C, the absorbance at 450 nm was spectrophotometrically measured.

### Colony formation assay

Cells were seeded in 6-well culture plates (2.5×10^3^ / well), the medium was replaced with fresh medium every two days, and the culture was terminated 7-10 days later. After the culture medium was removed, the cells were gently washed with PBS, fixed with methanol for 10 min, stained with 0.5% crystal violet for 30 min and washed with deionized water to remove the excess stain. Finally, the number of cell clones was counted.

### Conditioned medium collection

Cells were washed three times and replaced with serum-free medium. After 60 h, the medium was collected and centrifuged at 1,000 g for 10 min, 3,000 g for 10 min and 12,000 g for 30 min at 4 °C. The supernatant was fractioned at 3 KDa MWCO (Millipore) and filtered with a 0.22 μm filter (Millipore).

### Exosome extraction, identification and tracking analysis

Cells were cultured with fresh exosome-free medium at 37 °C for 48-72 h. Supernatants were collected and centrifuged at 1,000 g for 10 min at 4 °C, 12,000 g for 30 min at 4 °C, and at 110,000 g for 70 min at 4 °C. Then, the precipitates were washed with PBS and centrifuged at 110,000 g for 70 min at 4 °C. Finally, the precipitates were resuspended in PBS and filtered through a 0.22 μm filter. The exosomes were stored at -80 °C. Exosomal markers were detected by Western blot, and the morphology and structure of the exosomes were observed with NanoSight and transmission electron microscopy. Exosomes were stained with CellTracker™ CM-Dil Dye (C7001, Thermo Fisher Scientific) and visualized by Confocal laser scanning microscopy.

### Migration and invasion assays

The chambers (3422, Costar, Corning) were precoated with Matrigel (354248, Corning, USA) for the invasion assay. Cells were plated in the upper chamber with serum-free medium, whereas complete medium (10% FBS) was added to the bottom chamber. For the transwell assay using soluble chemoattractants, conditioned medium was placed into the bottom chamber, whereas cell suspension was plated in the upper chamber with serum-free medium. For the *in vitro* neutralization experiments, cells were plated in the upper chamber in serum-free medium containing CCL2 antibodies (mab479, R&D, 2 μg/mL), CXCL5 antibodies (mab433, R&D, 2 μg/mL), or isotype-matched control rat IgG2b antibodies (mab0061, R&D, 2 μg/mL). Complete or conditioned medium containing the corresponding antibodies was added to the bottom chamber. For the exosome treatment assays, the cells were incubated with exosomes for 48 h, and a transwell assay was performed. Cells were allowed to migrate and invade for 24-48 h, and cells in the upper chamber were fixed with methanol and stained with 0.5% crystal violet. Finally, the number of cells in four random microscopic fields was counted and averaged. The experiments were replicated three times. For the inhibitor treatment assays, cells were plated in the upper chamber in serum-free medium containing RAGE inhibitor FPS-ZM1 (HY-19370, MCE, 12 μM), CCR2 inhibitor RS102895 (HY-18611, MCE, 2 μM), or DMSO. Complete or conditioned medium containing the corresponding inhibitor was added to the bottom chamber.

### RNA-Seq

Total RNA was extracted with TRIzol Reagent (Life Technologies). Complementary DNA libraries were constructed using an Illumina TruSeq RNA Sample Prep kit according to the manufacturer's protocol. A total of 150 base paired-end reads were sequenced using the Illumina HiSeq 4000 platform in Mega Genomics. The read alignment was conducted using TopHat 2.0.13, and relative transcript abundances and differentially expressed genes were determined using the DESeq R package (1.36.0). Unsupervised clustering was performed using cluster and tree views. GO annotation and enrichment analyses were performed with differentially expressed genes (FDR < 0.01).

### Tandem mass tag quantitative proteomics

Conditioned medium was collected and condensed. The secreted protein quality was examined by SDS-PAGE. Proteins were pretreated and digested into peptides, then, the peptides were labeled using a TMT® Mass Tagging and Reagents kits (Pierce 90113, 90064). Proteins were identified and quantified by applying a Q Exactive mass spectrograph (Thermo Fisher Scientific). The raw data generated from the mass spectrometry were calculated and analyzed by utilizing the Proteome Discoverer software and mouse database (NCBI, txid_10090_mmu_76768_171213.fasta) with SEQUEST algorithm to identify differentially secreted proteins. Based on the KOBAS database, GO annotation and enrichment analyses were performed with differentially secreted protein. A protein interaction network diagram was constructed with the STRING database (http://string-db.org/) and drawn by Cytoscape software.

### Nuclear and cytoplasmic protein extraction

Nuclear and cytoplasmic proteins were extracted with an ExKine Nuclear and Cytoplasmic Protein Extraction kit (KTP3001, Abbkine) according to the manufacturer's protocol.

### Immunofluorescence

Cells were seeded on sterilized coverslips for 24 h. Cells were washed three times with PBS, fixed in 4% paraformaldehyde for 15 min and treated with 0.2% Triton X-100 for 5 min at room temperature. Then, the cells were incubated with 5% BSA for 1 h at room temperature, primary antibodies overnight at 4 °C, and fluorochrome-labeled secondary antibodies for 1 h at room temperature in the dark. Finally, the cells were washed with PBS, stained with DAPI and covered with coverslips and antifade mounting medium.

### Chromatin immunoprecipitation

ChIP assays were performed using a SimpleChIP® Plus Enzymatic Chromatin IP kit (9005, CST) with NF-κB antibody according to the manufacturer's instructions. The binding of NF-κB in the promoter region of CCL2 or CXCL5 gene was detected by qPCR. The primers are listed in **Table S1**.

### Caffeic acid phenethyl ester (CAPE) treatment

Cells were seeded in 6-well culture plates and the medium was replaced with fresh medium containing CAPE (2 μM, S7414, Selleck). Cells were harvested 48 h later.

### Animals

BALB/c mice, BALB/c nude mice and SCID beige mice were purchased from Beijing Vital River Laboratory Animal Technology Co., Ltd. S100A14^flox/flox^ C57BL/6J mice were generated by Cyagen Biosciences Inc. CMV-Cre C57BL/6J mice and PyMT C57BL/6J mice were obtained from the Model Animal Research Center of Nanjing University. S100A14^-/-^ mice were generated by crossing S100A14^flox/flox^ mice with CMV-Cre mice, then S100A14^-/-^ PyMT mice were acquired by several steps of crossing S100A14^-/-^ mice with PyMT mice.

### Animal studies

All animal procedures were reviewed and approved by the Institutional Animal Care and Use Committee of the Chinese Academy of Medical Sciences Cancer Hospital. For spontaneous lung metastasis experiments, 4T1 cells (2×10^5^/100 μL) were injected into the mammary fat pad of female BALB/c mice (6-8 weeks). For the exosome treatment assays, 4T1 cells and 4T1-derived exosomes were simultaneously injected into the mammary fat pads of female BALB/C mice. For the *in vivo* neutralization experiments, 4T1 cells were injected as described above. Three days later, the mice were randomly assigned to treatment groups and control groups that all received intraperitoneal injections of an isotype-matched control rat IgG2b antibody (mab0061, R&D, 5 mg/kg), a CXCL5 antibody (mab433, R&D, 5 mg/kg) or a CCL2 antibody (mab479, R&D, 5 mg/kg). Antibody treatments were conducted once per week for 4 weeks in succession. Spontaneous lung metastasis was also observed in female wild-type and S100A14^-/-^ C57BL/6J mice carrying PyMT. For experimental metastasis assays, MDA-MB-231 cells (1×10^6^/100 μL) and MCFCA1a cells (5×10^5^/100 μL) were injected into the tail veins of SCID beige mice (6-8 weeks) and BALB/c female nude mice (6-8 weeks), respectively. Tumor growth was monitored weekly, at the end of the experiments, mice were euthanized, the tumors were weighed, and pulmonary metastasis nodules were analyzed. For TAM depletion experiments, control liposome and liposomal clodronate (CLD-8901, Encapsula NanoSciences) were injected intraperitoneally at day 1, then the orthotopic mammary fat pad transplantation of 4T1 cells was performed next day and followed by intraperitoneal injection of clodronate encapsulated in liposomes for macrophage depletion. Liposomes treatments were conducted once per week for 4 weeks in succession. 4 weeks later, the mice were euthanized and the lung tissues were dissected and metastasis nodules were calculated.

### ELISA

The protein levels of IL1A, CCL2, CXCL3, and CXCL5 in the conditioned medium were detected by IL1A (DY400, R&D), CCL2 (DY479, R&D), CXCL3 (EK1364, BOSTER) and CXCL5 (EK0919, BOSTER) ELISA kits. The serum S100A14, CCL2 and CXCL5 levels in breast cancer patients and healthy controls were examined using S100A14 (QS440428, Beijing Qisong Biotechnology Co., Ltd.), CCL2 (QS40081, Beijing Qisong Biotechnology Co., Ltd.), and CXCL5 (QS41512, Beijing Qisong Biotechnology Co., LTD) ELISA test kits.

### Immunohistochemistry analysis

The tissues embedded in paraffin were cut into 4 μm thick sections and then deparaffinized. Then, 3% hydrogen peroxide was added and incubated with the sections at room temperature for 30 min to eliminate endogenous peroxidase activity. The sections were immersed in citrate buffer (pH 6.0) and heated for 2 min using a pressure cooker for antigen retrieval. The tissues were blocked with goat serum at 37 °C for 1 h. The samples were incubated with primary antibodies overnight at 4 °C. Then, the samples were incubated with a second antibody at room temperature for 1 h. LP staining was performed using a PV-9000 polymer detection system for IHC staining kit (ZSGB-BIO, Beijing, China). The staining intensity was graded from 0 to 3, with no staining scored as 0, weak positive staining as 1, positive staining as 2, and strong positive staining as 3. The percentage of staining was automatically evaluated by ImageScope software (Aperio Technology). The expression score was determined by the staining intensity and the percentage of staining. The number of F4/80^+^ macrophages in three random microscopic fields was counted and averaged. The antibodies used are listed in **Table S3**.

### Clinical specimens

For the immunochemical analysis, patients with histologically confirmed breast cancer who had received no prior treatment were recruited at Cancer Hospital, Harbin Medical University. The primary breast cancer tissues and paired adjacent normal tissues, metastatic lymph node tissues and non-metastatic lymph node tissues were collected. Informed consent was obtained from all subjects, and this study was approved by the ethical committees of Harbin Medical University. The serum samples from breast cancer patients and healthy controls were collected at Cancer Hospital, Chinese Academy of Medical Sciences. Informed consent was obtained from all subjects, this study was approved by the Institutional Review Board of the National Cancer Center/Cancer Hospital, Chinese Academy of Medical Sciences.

### Statistical analysis

Statistical analyses were performed using GraphPad Prism 7 and SPSS 24.0. All data are presented as the mean ± SD unless otherwise stated. Student's *t*-test was used to determine the significance unless otherwise stated (Spearman's correlation). *P* < 0.05 was considered statistically significant.

## Results

### S100A14 promotes cell migration and invasion *in vitro* and lung metastasis *in vivo* in breast cancer

**Figure 1:**
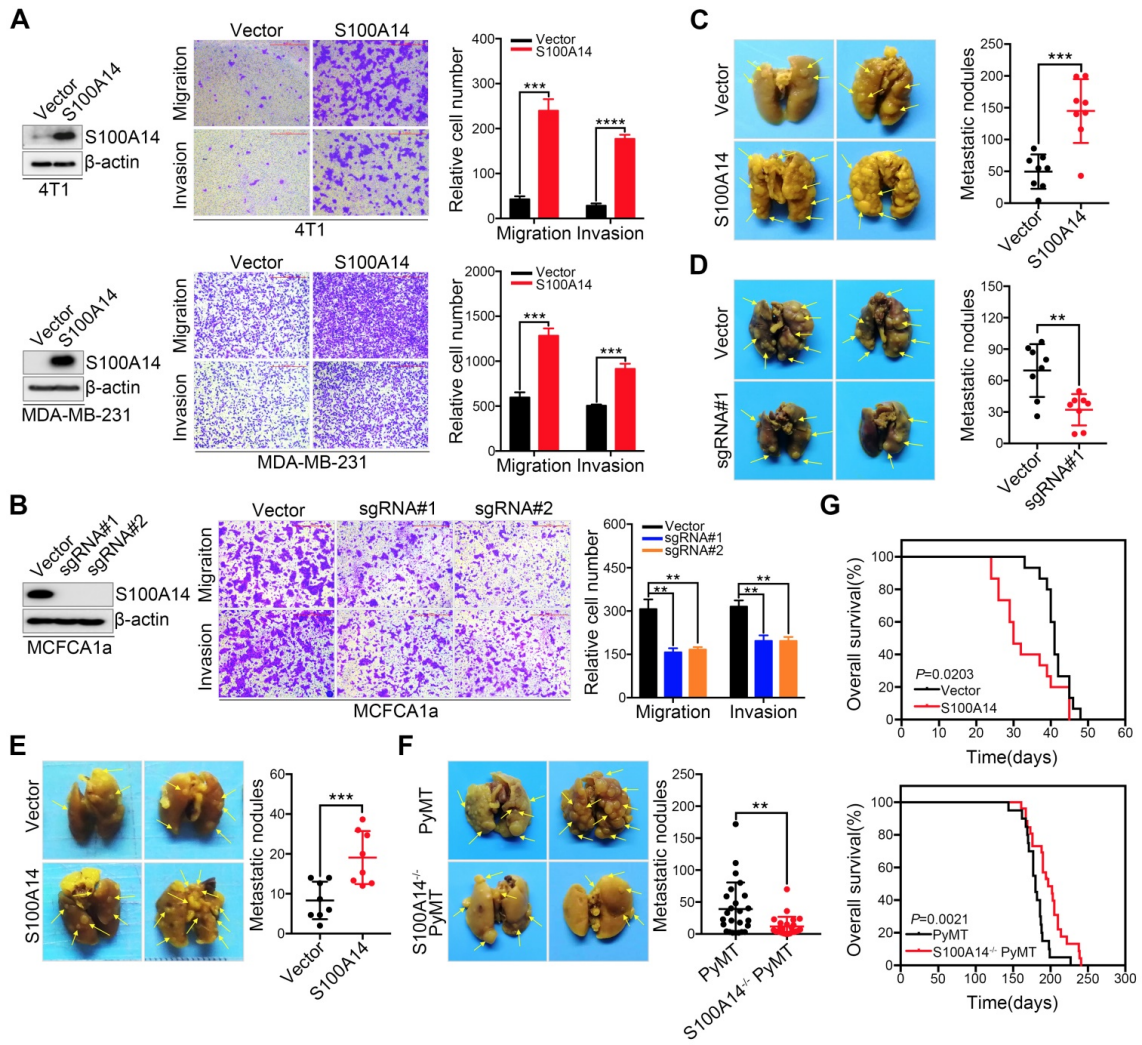
** S100A14 facilitates breast cancer cell migration and invasion *in vitro* and lung metastasis *in vivo*. (A) Left**, the overexpression efficiency of S100A14 in 4T1 (**upper**) and MDA-MB-231 (**lower**) cells was determined by Western blot. **Right**, representative images and quantitative analysis of migration and invasion assays in 4T1 (**upper**) and MDA-MB-231 (**lower**) cells. Scale bars=500 μm. **(B) Left**, the S100A14 knockout efficiency was determined by Western blot. **Right**, representative images and quantitative analysis of migration and invasion assays in MCFCA1a cells. Scale bars=500 μm. **(C)** S100A14-overexpressing MDA-MB-231 and control cells were injected into SCID beige through the tail vein. Representative images and statistical analyses of lung metastatic nodules are shown (n=8). **(D)** S100A14-knockout MCFCA1a and control cells were injected into BALB/c nude mice through the tail vein. Representative images and statistical analyses of lung metastatic nodules are shown (n=8). **(E)** S100A14-overexpressing 4T1 and control cells were injected into the mammary fat pads of BALB/c mice. Representative images and statistical analyses of lung metastatic nodules are shown (n=8). **(F)** Lung metastatic nodules of mice were analyzed at week 22 from birth in PyMT (n=25) and S100A14^-/-^ PyMT (n=25) mice. Representative images and statistical analyses of lung metastatic nodules are shown. **(G)** Kaplan-Meier curve of mice (**upper**) injected with S100A14-overexpressing 4T1 (n=15) and control cells (n=15) and S100A14^-/-^ PyMT (n=26) and PyMT (n=20) mice (**lower**). The horizontal axis is the survival day, and the vertical axis is the percentage of surviving cases among total cases. The log-rank test *P* value is displayed on the graph. Data in **A** and **B** are presented as the mean of biological replicates in a representative experiment ± SD. Data in **C**-**F** are presented as the mean ± SD. Data were analyzed by an unpaired two-tailed Student's *t*-test. **P<0.01, ***P<0.001, ****P<0.0001.

We previously reported that S100A14 enhances breast cancer cell migration and invasion, prompting us to further explore the role of S100A14 in the metastasis of breast cancer in the physiological context. First, we screened the expression of S100A14 in nine breast cancer cell lines and normal breast epithelial cells by Western blot (**Figure S1A**). We established stable S100A14-overexpressing cells using retroviral- and lentiviral-mediated gene delivery in 4T1 and MDA-MB-231 cells with very low basal expression levels of S100A14. Furthermore, S100A14-knockout cells were generated using the CRISPR-Cas9 system in MCFCA1a cells with a high endogenous S100A14 expression level. S100A14 expression was detected by qRT-PCR and Western blot (**Figure S1B**, **Figure 1A-B left panel**). We next determined the effect of S100A14 on cell migration and invasion by transwell assay. As expected, the overexpression of S100A14 dramatically promoted the migration and invasion of 4T1 and MDA-MB-231 cells, whereas S100A14 ablation suppressed the migration and invasion of MCFCA1a cells (**Figure 1A-B right panel**). To further determine whether S100A14 promotes metastasis *in vivo*, we injected S100A14-overexpressing MDA-MB-231 cells, S100A14-knockout MCFCA1a cells and the corresponding control cells into severe combined immunodeficiency (SCID) beige and BALB/c nude mice via the tail vein. In accordance with the *in vitro* results, S100A14 overexpression significantly augmented the accumulation of macrometastases in the lungs of immune-deficient mice, and S100A14 ablation decreased pulmonary metastasis (**Figure 1C-D**). The orthotopic mammary fat pad transplantation of 4T1 cells further validated that S100A14 enhances breast cancer cell metastasis (**Figure 1E**). To determine whether S100A14 ablation delays the development of metastasis in the mouse model, we crossed S100A14-null (S100A14^-/-^) mice with luminal B breast cancer model MMTV-PyMT (PyMT) mice to generate spontaneous breast tumors that lack S100A14 expression (S100A14^-/-^ PyMT). As anticipated, in the context of PyMT, S100A14-deficient tumors had a much lower propensity to metastasize to the lung than their S100A14 wild-type (WT) counterparts (**Figure 1F**). Remarkably, the overall survival analysis demonstrated that S100A14 overexpression is associated with a reduced overall survival time (**Figure 1G upper panel**). In contrast, the loss of S100A14 corresponds to an increased overall survival time (**Figure 1G lower panel**). The well-characterized function of S100A14 is the regulation of cell proliferation 18, 19. To exclude the possibility that the effect of S100A14 on metastasis is due to increased cell proliferation, CCK-8 assay, colony formation assay and xenograft experiments were performed with S100A14-overexpressing 4T1 cells and their corresponding control cells. The results showed that S100A14 had no significant influence on breast cancer cell proliferation (**Figure S1C-D**). Consistently, the loss of S100A14 in PyMT mice had no effect on the number and diameter of tumors (**Figure S1E**). Collectively, these results strongly indicate that S100A14 facilitates breast cancer metastasis and drives poor prognosis.

### Exosomes containing S100A14 exert a metastasis-promoting effect in breast cancer

**Figure 2:**
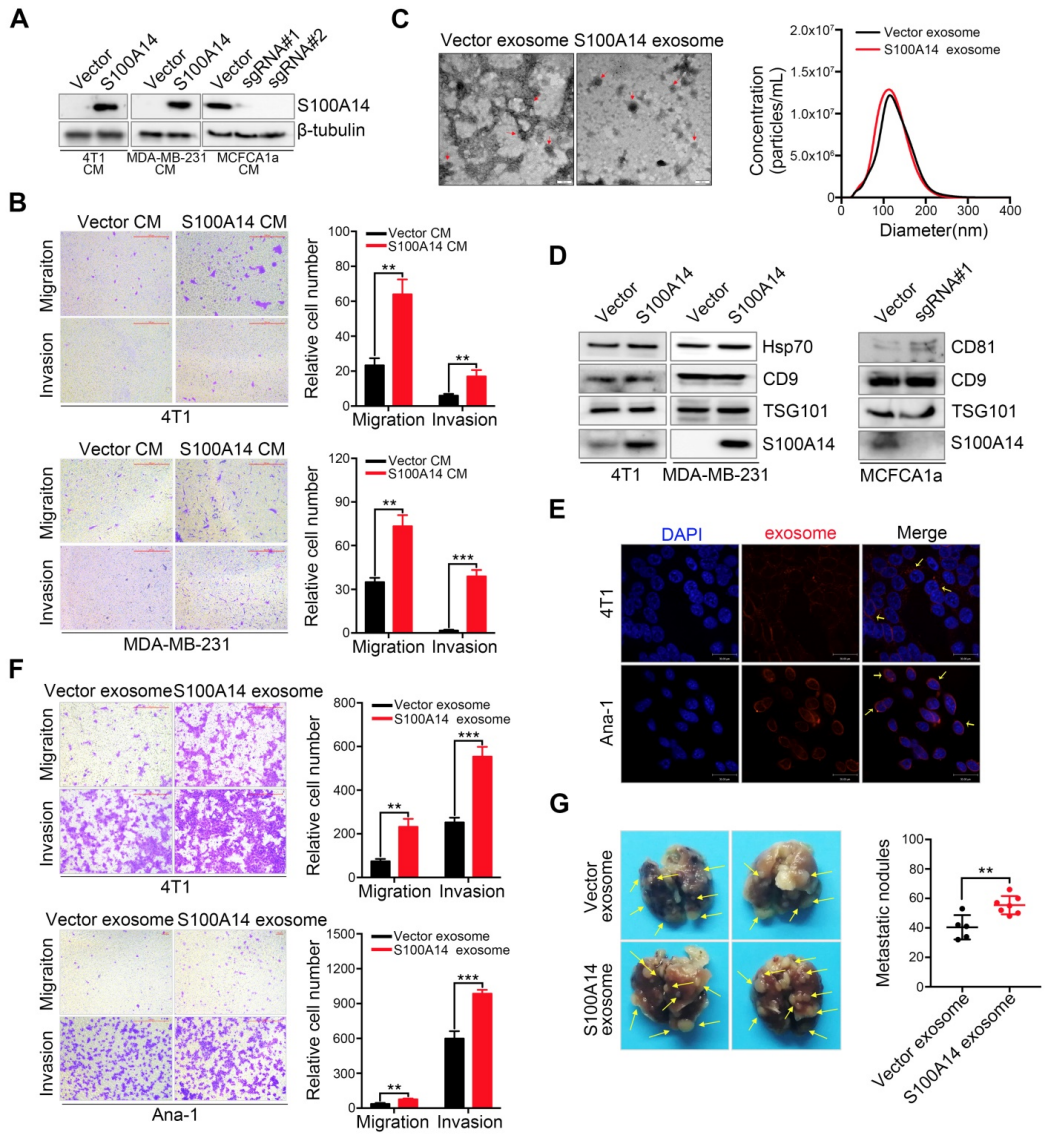
** Exosomes containing S100A14 promote breast cancer metastasis**.** (A)** The protein level of S100A14 in conditioned medium derived from S100A14-overexpressing 4T1, MDA-MB-231 and S100A14-knockout MCFCA1a cells and their corresponding control cells was detected by Western blot. **(B)** Transwell assays with 4T1 (**upper**) and MDA-MB-231 (**lower**) cells were performed with the corresponding condition medium as a chemoattractant. Representative images and quantitative analysis of migration and invasion are shown. Scale bars=500 μm. **(C) Left**, transmission electron micrograph of S100A14-overexpressing 4T1 cell exosomes and control cell exosomes. Gold particles are depicted as red dots, scale bars=100 nm. **Right,** exosome concentrations and particle size distributions were accurately measured by NanoSight.** (D)** An immunoblot of the exosomal markers Hsp70, TSG101, CD9, CD81 and S100A14 is shown. **(E)** Exosomes were stained with CellTracker™ CM-Dil Dye, and then Dil-labeled exosomes were co-incubated with 4T1 and Ana-1 cells. Confocal laser scanning microscopy was used to observe whether exosomes retained their activity and could enter 4T1 and Ana-1 cells. Scale bars=30 μm. **(F)** 4T1 (**upper**) and Ana-1 (**lower**) cells were treated with exosomes derived from S100A14-overexpressing 4T1 and control cells, and migration and invasion assays were performed. Representative images and quantitative analyses are shown. Scale bars=100 or 500 μm. **(G)** 4T1 cells and exosomes derived from S100A14-overexpressing 4T1 and control cells were co-injected into the mammary fat pads of BALB/c mice (n=5, 7), and lung metastatic nodules were examined after 5 weeks. Representative images (**Left**) and statistical analyses of lung metastatic nodules (**Right**) are shown. Data in **B** and **F** are presented as the mean of biological replicates in a representative experiment ± SD. Data in **G** are presented as the mean ± SD. Data were analyzed by an unpaired two-tailed Student's *t*-test. **P<0.01, ***P<0.001.

A unique characteristic of certain S100 proteins is their occurrence in extracellular spaces and even secretion in the serum, we therefore examined the secretion of S100A14 in the extracellular medium. A marked increase in S100A14 secretion was found in S100A14-overexpressing breast cancer cells, whereas a striking reduction was observed in S100A14-knockout MCFCA1a cells (**Figure 2A**). Next, we performed transwell assays to examine the effects of secreted S100A14 on cell migration and invasion. Notably, the conditioned medium from S100A14-overexpressing cells significantly enhanced the migration and invasion of breast cancer cells (**Figure 2B**). Recently, exosomes have attracted attention as important mediators of intercellular communication 20. Exosomes containing proteins and nucleic acids play an important role in the horizontal transfer of proteomic and genetic information to target cells 21. Several studies have shown that tumor-derived exosomes can facilitate tumor progression and metastasis 22-24, we then assessed whether S100A14 protein is present in cancer cell exosomes. Exosomes derived from S100A14-overexpressing 4T1 cells and vector control cells were isolated using established ultracentrifugation methods. The harvested exosomes were analyzed by transmission electron microscopy (**Figure 2C**, **left panel**) and NanoSight (**Figure 2C**, **right panel**). The identity of the exosomes was confirmed through the detection of Hsp70, TSG101, CD9 and CD81 exosomal markers, and S100A14 protein was also detected in the isolated exosomes. (**Figure 2D**). And more studies demonstrate that TAMs can promote the development, malignant progression and metastasis of breast cancer, and is associated with poor prognosis of patients. To explore the effect of the supernatant of 4T1 murine breast cancer cell on Ana-1 macrophages *in vitro* by simulating the microenvironment of breast cancer, we selected Ana-1 cells to perform *in vitro* experiment. 4T1 and Ana-1 cells were labeled with CellTracker™ CM-Dil Dye, and the fluorescence signal was detected (**Figure 2E**). Transwell assays showed that exosomes derived from S100A14-overexpressing cells significantly promoted 4T1 and Ana-1 cell migration and invasion (**Figure 2F**). An* in vivo* experimental metastasis assay further validated the promoting effect on metastasis by exosomes derived from S100A14-overexpressing cells compared to control cells (**Figure 2G**). These results suggest that S100A14 is present in exosomes and exerts a metastasis-promoting function.

### S100A14 promotes the expression of CCL2/CXCL5 via NF-κB-mediated transcriptional activation

To uncover the molecular mechanisms by which S100A14 promotes breast cancer metastasis, we performed RNA-Seq and quantitative proteomics analyses of secreted proteins in S100A14-overexpressing 4T1 cells and control cells.

**Figure 3:**
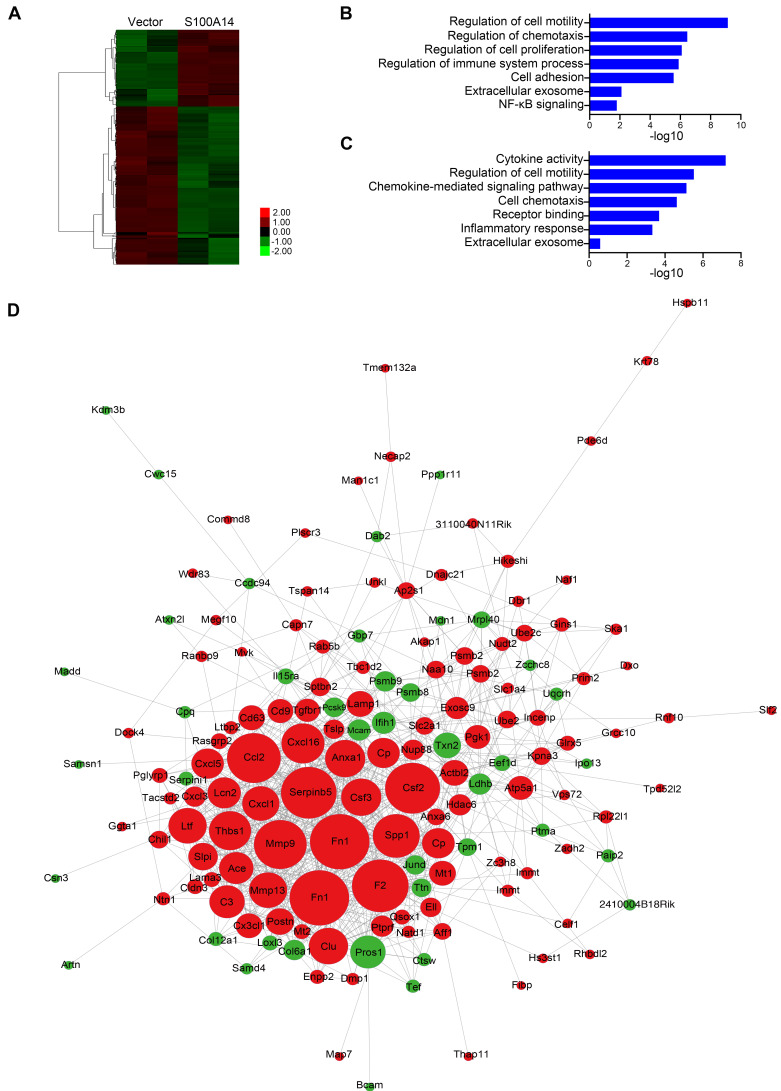
** S100A14 regulates the expression of cytokines and chemokines**.** (A)** Heatmap showing the unsupervised clustering of differentially expressed genes regulated by S100A14. **(B)** Functional enrichment analysis of the differentially expressed genes regulated by S100A14.** (C)** Functional enrichment analysis of the differentially secreted proteins regulated by S100A14.** (D)** Interactive network diagram of the differentially secreted proteins regulated by S100A14.

**Figure 4:**
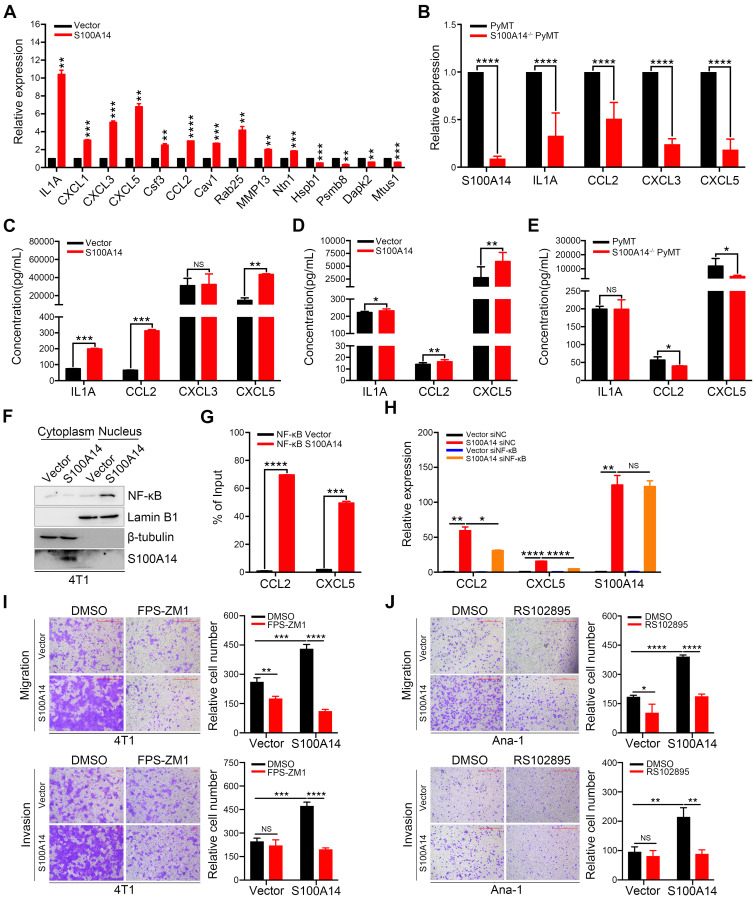
** S100A14 augments the expression and secretion of cytokines and chemokines**. **(A)** The mRNA expression of candidate genes was detected by qRT-PCR. **(B)** The expression of S100A14, IL1A, CCL2, CXCL3 and CXCL5 in tumor tissues derived from S100A14^-/-^ PyMT and PyMT mice was detected by qRT-PCR.** (C)** The levels of IL1A, CCL2, CXCL3 and CXCL5 in the conditioned medium from S100A14-overexpressing 4T1 and control cells were examined by ELISA.** (D)** The serum levels of IL1A, CCL2 and CXCL5 in mice injected with S100A14-overexpressing 4T1 and control cells were examined by ELISA.** (E)** The serum levels of IL1A, CCL2 and CXCL5 in S100A14^-/-^ PyMT and PyMT mice were examined by ELISA. **(F)** The expression of NF-κB p65 was determined by Western blot in the fractionated lysates of S100A14-overexpressing 4T1 and control cells. Lamin B1 and β-tubulin were used as loading controls for nuclear and cytosolic fractions, respectively. **(G)** ChIP for endogenous NF-κB p65 in S100A14-overexpressing 4T1 and control cells, followed by qPCR at the promoter region of CCL2 and CXCL5. (**H**) S100A14-overexpressing 4T1 and control cells were transfected with 60 nM NF-κB siRNA and control siRNA. After 48 h, the expression of S100A14, CCL2 and CXCL5 was detected by qRT-PCR. (**I**) Transwell assays were performed in S100A14-overexpressing 4T1 and control cells treated with FPS-ZM1 for 24 h. Representative images and statistical analyses are shown. Scale bars=500 μm or 100 μm. **(J)** Ana-1 cells were placed into the upper chamber; conditioned medium which was derived from S100A14-overexpressing 4T1 and control cells was mixed with RS102895 and placed into the lower chamber and transwell assays were performed for 24 h. Representative images and statistical analyses are shown. Scale bars=500 μm. Data in** A-J** are presented as the mean ± SD. Data were analyzed by an unpaired two-tailed Student's t-test. *P<0.05, **P<0.01, ***P<0.001, ****P<0.0001. NS means no significant difference.

Using differential expression analysis (DEseq; FDR < 0.01), we identified 66 genes with upregulated expression and 136 genes with downregulated expression in response to S100A14 overexpression. A heatmap showing unsupervised clustering of expression Z-scores of 202 differentially expressed genes is shown in **Figure 3A** and **Table S4**. Gene Ontology (GO) enrichment analysis revealed that the differentially expressed genes were significantly enriched in GO terms related to the regulation of cell motility and chemotaxis (**Figure 3B**). Consistently, mass spectrometry analysis of the secreted proteins showed that the overexpression of S100A14 facilitated the secretion of cytokines and chemokines and led to significant enrichment of proteins associated with cell chemotaxis and cell mobility (**Figure 3C**). Remarkably, the interactive network analysis revealed that S100A14 upregulated the expression of a series of cytokines and chemokines located at the core of the network (**Figure 3D**, **Table S4**). Consistent with these findings, qRT-PCR analysis further confirmed that the expression of IL1A, CCL2, CXCL1, CXCL3, CXCL5, Cav1, Csf3, Ntn1, Rab25 and MMP13 was upregulated after S100A14 overexpression (**Figure 4A**). Conversely, the mRNA expression of S100A14, IL1A, CCL2, CXCL3 and CXCL5 in tumor tissues derived from S100A14-deficient mice was significantly reduced compared with those from their WT counterparts (**Figure 4B**).

ELISA experiments demonstrated that the overexpression of S100A14 boosted the secretion of IL1A, CCL2 and CXCL5 in the conditioned medium (**Figure 4C**). We further measured the serum levels of IL1A, CCL2 and CXCL5 collected from 4T1 orthotopically implanted BALB/c mice. The results illustrated that the serum levels of CCL2 and CXCL5 were increased in mice transplanted with S100A14-overexpressing 4T1 cells compared to mice transplanted with control cells, whereas a slight increase in the serum IL1A level was found (**Figure 4D**). Correspondingly, the loss of S100A14 significantly reduced the serum concentration of CCL2 and CXCL5, and no change was observed in the serum IL1A concentration (**Figure 4E**). Thus, these results suggest that S100A14 increases the expression and secretion of cytokines and chemokines. Most notable change among these cytokines and chemokines was the prominent induction of CCL2 and CXCL5. We next sought to unveil the mechanisms by which S100A14 enhances CCL2 and CXCL5 expression. Several members of the S100 calcium-binding protein family have been reported to exert their effects via the RAGE-mediated activation of the NF-κB signaling pathway 25, 26. Strikingly, the transcription factor NF-κB has previously been shown to transcriptionally regulate CCL2 and CXCL5 expression 27, 11. These studies prompted us to explore whether S100A14 induces CCL2 and CXCL5 expression through NF-κB-mediated transcriptional regulation. We first analyzed NF-κB localization in the fractionated lysates from S100A14-overexpressing cells and control cells. As anticipated, S100A14 overexpression resulted in an increase in nuclear NF-κB with a parallel decrease in the cytoplasm (**Figure 4F**). Immunofluorescence assays further validated that S100A14 promoted the translocation of NF-κB from the cytoplasm to the nucleus (**Figure S2A**). Next, we performed a ChIP assay to determine whether S100A14 induces the binding of NF-κB to the regulatory regions of CCL2 and CXCL5. Indeed, the ChIP analysis demonstrated dramatically increased enrichment of NF-κB at the consensus sequence within the CCL2 and CXCL5 promoters in S100A14-overexpressing 4T1 cells compared to control cells (**Figure 4G**). Most importantly, we confirmed that the S100A14-induced expression of CCL2 and CXCL5 was significantly suppressed following by NF-κB inhibition using siRNA or NF-κB-specific inhibitor CAPE treatment (**Figure 4H**, **Figure S2B-C**). Our previous study has shown that S100A14 can interact with RAGE 28, to determine whether RAGE is a functional receptor of S100A14 activating NF-κB, we performed transwell assay in S100A14-overexpressed 4T1 and control cells treated with RAGE inhibitor-FPS-ZM1. FPS-ZM1 treatment obviously suppressed cell migration and invasion in S100A14-overexpressed 4T1 cells. In contrast, no effect on cell invasion and a slight effect on cell migration was observed in control cells. These results suggest that S100A14 can exert its metastasis-promoting function via the RAGE in the tumor cells (**Figure 4I**). Also, we investigated the role of the receptor of CCL2-CCR2 in S100A14-induced cell migration and invasion 29. We performed transwell assays in Ana-1 cells with conditional medium derived from S100A14-overexpressed 4T1 and control cells which was mixed with CCR2 inhibitor-RS102895. RS102895 treatment significantly decreased cell migration and invasion in Ana-1 cells treated with conditional medium derived from S100A14-overexpressed 4T1 cells, while no significant or a slight effect was observed on control cells, suggesting that S100A14 can promote macrophage migration and invasion via the CCL2-CCR2 axis (**Figure 4J**). Collectively, our findings imply that S100A14 activates NF-κB-dependent transcription on CCL2 and CXCL5 via RAGE and establish a clear link among S100A14, RAGE, NF-κB and CCL2/CXCL5.

### S100A14-CCL2/CXCL5 axis acts as a metastasis driver in breast cancer

**Figure 5:**
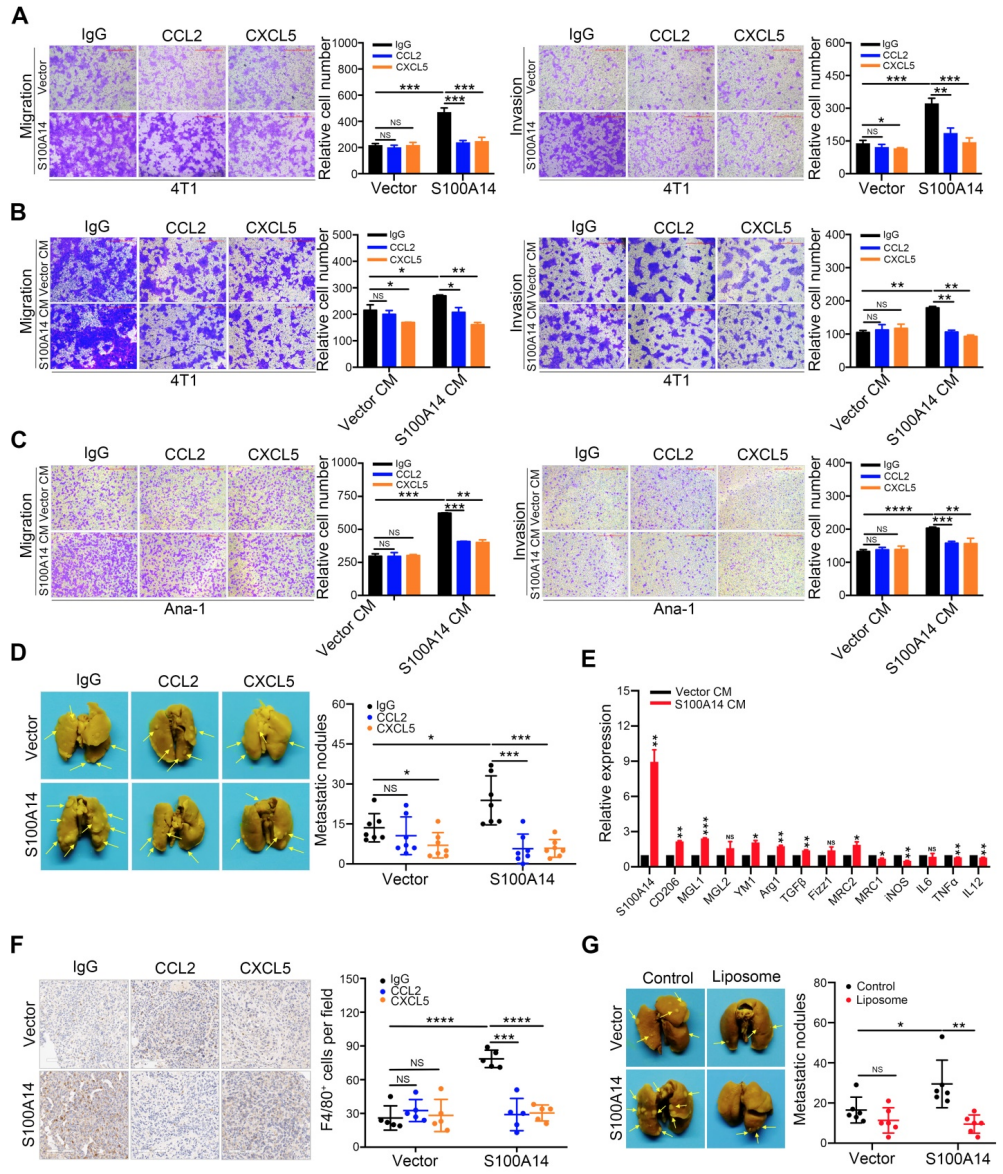
** The S100A14-CCL2/CXCL5 axis drives metastasis in breast cancer**.** (A)** S100A14-overexpressing 4T1 and control cells were pretreated with CCL2- and CXCL5-neutralizing antibodies for 24 h, transwell assays were performed. Representative images and statistical analyses are shown. Scale bars=500 μm. **(B)** Conditioned medium was pretreated with CCL2- and CXCL5-neutralizing antibodies for 48 h; 4T1 cells were placed into the upper chamber; conditioned medium derived from S100A14-overexpressing 4T1 and control cells was placed into the lower chamber and transwell assays were performed. Representative images and statistical analyses are shown. Scale bars=500 μm. **(C)** Conditioned medium was pretreated with CCL2- and CXCL5-neutralizing antibodies for 48 h. Ana-1 cells were placed into the upper chamber, conditioned medium derived from S100A14-overexpressing 4T1 and control cells was placed into the lower chamber. Transwell assays were performed. Representative images and statistical analyses are shown. Scale bars=500 μm. **(D)** S100A14-overexpressing 4T1 cells and control cells were injected into the mammary fat pads of BALB/c mice. After 3 days, CCL2- and CXCL5-neutralizing antibodies were injected intraperitoneally once a week. After 4 weeks, mice were euthanized and dissected. Representative images (**left**) and statistical analyses of lung metastatic nodules (**right**) are shown (n=7). **(E)** Ana-1 cells were treated with conditioned medium derived from S100A14-overexpressing 4T1 cells and control cells for 48 h. The expression of M1 and M2 macrophage markers was detected by qRT-PCR. **(F)** The number of invaded macrophages was analyzed in lung tissues by IHC. Representative images (**left**) and statistical analyses (**right**) are shown (n=5). Scale bars=100 μm.** (G)** Control liposome and liposomal clodronate were injected intraperitoneally at day 1, then S100A14-overexpressing 4T1 and control cells were injected into the mammary fat pads of BALB/c mice at next day. Control liposome and liposomal clodronate were injected intraperitoneally once a week. After 4 weeks, mice were euthanized and dissected. Representative images (**left**) and statistical analyses of lung metastatic nodules (**right**) are shown (n=6). Data in **A-C** are presented as the mean of biological replicates in a representative experiment ± SD. Data in **D-G** are presented as the mean ± SD. Data were analyzed by an unpaired two-tailed Student's *t*-test. ^*^P<0.05, **P<0.01, ***P<0.001, ****P<0.0001. NS means no significant difference.

To directly test the potential contribution of CCL2 and CXCL5 to S100A14-induced migration, invasion and metastasis, CCL2- and CXCL5-neutralizing antibodies were used. Strikingly, the blocking of CCL2 and CXCL5 significantly inhibited the S100A14-induced migration and invasion of 4T1 cells (**Figure 5A**). To further examine whether CCL2 and CXCL5 are essential for S100A14-induced cell migration and invasion, 4T1 cells were incubated with conditioned medium derived from S100A14-overexpressing 4T1 cells and control cells pretreated with CCL2- and CXCL5-neutralizing antibodies in a transwell assay. The results showed that CCL2 and CXCL5 neutralization abolished the increased migration and invasion by S100A14 (**Figure 5B**). Given the critical roles of CCL2 and CXCL5 in recruiting TAMs to neoplastic tissue, thereby contributing to cancer metastasis 30, 31, we investigated whether S100A14 plays a key role in orchestrating the recruitment of macrophages through CCL2 and CXCL5. The transwell assay results suggested that the conditioned medium from S100A14-overexpressing 4T1 cells strengthened the migration and invasion of Ana-1 cells compared to that from control 4T1 cells. When CCL2 and CXCL5 were blocked with neutralizing antibodies, the increased migration and invasion caused by S100A14 was inhibited (**Figure 5C**). To further confirm whether CCL2 and CXCL5 function as pivotal chemokines mediating S100A14-induced breast cancer metastasis *in vivo*, we used the 4T1 orthotopic transplantation model in which mice were treated with CCL2- and CXCL5-neutralizing antibodies or IgG control antibodies.

As expected, mice transplanted with S100A14-overexpressing 4T1 cells showed significantly increased lung metastasis compared with mice transplanted with control cells, and the inhibition of CCL2/CXCL5 was sufficient to diminish the S100A14-induced lung metastasis to a level comparable with that of the control *in vivo* (**Figure 5D**). The transition of macrophages from the M1 to M2 phenotype is crucial for tumor metastasis 32-34. We examined the expression of M1 and M2 macrophage markers in Ana-1 cells treated with conditioned medium derived from S100A14-overexpressing 4T1 cells and control cells. In parallel with the decrease in the expression of the M1 markers iNOS, IL12, MRC1 and TNFα, M2 marker Arg1, TGFβ, CD206, MGL1, MRC2 and YM1 expression was induced by S100A14 overexpression (**Figure 5E**). Furthermore, we performed IHC analysis using F4/80 antibody to determine the effects of the S100A14-CCL2/CXCL5 axis on macrophage recruitment in the mouse metastatic lung tissues. We observed a significantly increased number of macrophages in metastatic lung tissues overexpressing S100A14. Accordingly, the suppression of CCL2 and CXCL5 reduced the S100A14-induced increase in the number of macrophages (**Figure 5F**). To further confirm whether S100A14-induced metastasis is dependent on TAMs, the orthotopic mammary fat pad transplantation of 4T1 cells was performed and followed by intraperitoneal injection of clodronate encapsulated in liposomes for macrophage depletion. And the results demonstrated that S100A14 enhanced breast cancer metastasis, but S100A14-promoting metastasis was blocked by TAM depletion with Liposomal Clodronate (**Figure 5G**). Thus, these results imply that S100A14 enhances breast cancer cell and macrophage migration and invasion via CCL2 and CXCL5, and the S100A14-CCL2/CXCL5 axis acts as a driver of breast cancer metastasis.

### Increased expression of S100A14, CCL2 and CXCL5 correlates with poor survival of breast cancer patients

**Figure 6:**
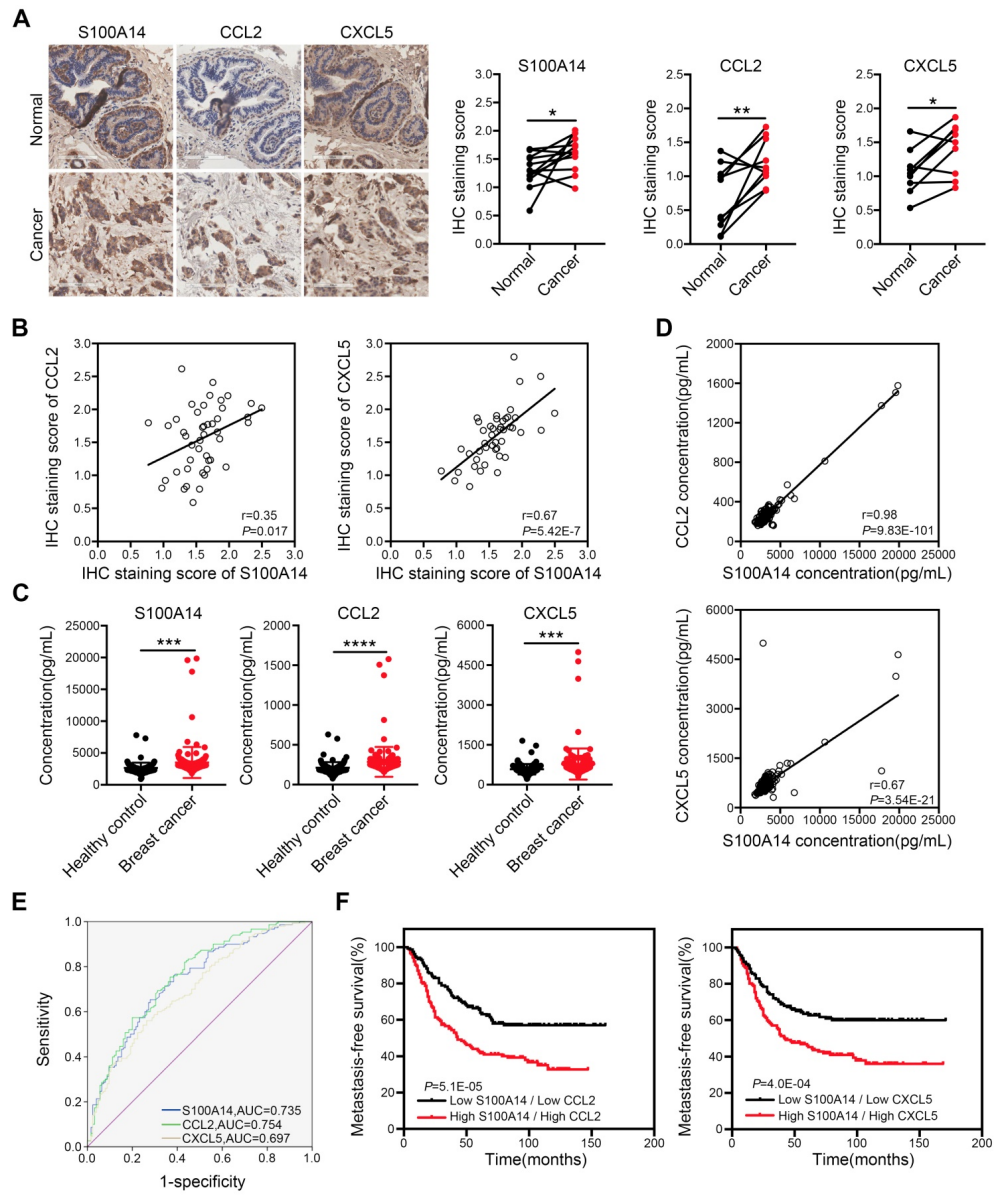
** Clinical significance of S100A14, CCL2 and CXCL5 in breast cancer**.** (A)** The expression of S100A14, CCL2 and CXCL5 was analyzed in breast cancer tissues and their adjacent normal tissues by IHC. Representative images (**left**) and statistical analyses (**right**) showing by IHC staining that S100A14, CCL2 and CXCL5 are overexpressed in breast cancer tissues. Scale bars=100 μm. **(B)** Pearson's correlation analysis showing that S100A14 expression significantly correlates with CCL2 and CXCL5 expression. **(C)** Serum levels of S100A14, CCL2 and CXCL5 in breast cancer patients and healthy controls were detected by ELISA. The statistical analysis of S100A14, CCL2 and CXCL5 serum levels in breast cancer patients and healthy controls is shown. **(D)** Pearson's correlation analysis showing that the S100A14 level significantly correlates with the CCL2 and CXCL5 in serum levels. **(E)** The ROC curve of S100A14, CCL2 and CXCL5 serum levels of breast cancer patients and healthy controls was analyzed to calculate the optimal sensitivity and specificity for the detection of breast cancer. **(F)** Kaplan-Meier analysis for metastasis-free survival of breast cancer patients in the GEO database (GSE2034, GSE2603, GSE5327 and GSE12276). Patients were divided into two groups based on the combined analysis according to the median value for S100A14 and CCL2/CXCL5. The log-rank test *P* values are shown. Data in **A** is statistically analyzed with paired *t*-test; and **C** are presented with the mean ± SD and analyzed by 2-sided *t*-test; * P<0.05, ** P<0.01, *** P<0.001, **** P<0.0001.

To define the physiological significance of these observations, we first analyzed the expression of S100A14 in 233 paired breast cancer specimens and adjacent normal tissues, 177 cases of lymph node specimens with metastasis and 174 cases of lymph node specimens without metastasis by IHC. The results demonstrated that S100A14 protein was mainly localized to the cell membrane and the cytoplasm. S100A14 expression was obviously upregulated in breast cancer tissues compared with the matched normal tissues (**Figure S3A**). Additionally, the S100A14 expression increase corresponded with the lymph node metastasis (**Figure S3B**). Gene amplification is the most frequently identified type of genetic change associated with cancer. It has been reported that the amplification of chromosome 1q21.3, which includes S100 calcium-binding proteins, is highly enriched in tumor-initiating cells (TICs) and recurrent tumors in breast cancer 16. To test whether the upregulation of S100A14 expression is associated with copy number amplification in breast cancer, we analyzed S100A14 copy number alterations using breast cancer data from The Cancer Genome Atlas (TCGA). The results showed that copy number amplification of S100A14 was present in 5.4%-20.7% of primary breast cancer patients, but S100A14 amplification was found in approximately 26.1% of metastatic breast cancer patients (**Figure S3C**). Remarkably, the copy number amplification was significantly correlated with the increased S100A14 mRNA expression (**Figure S3D**). Correspondingly, the TCGA database analysis demonstrated that S100A14 expression is significantly associated with lymph node metastasis (**Figure S3E**). Together, these results suggest that the S100A14 copy number is amplified, it is overexpressed, and this overexpression is associated with lymph node metastasis in breast cancer. To further investigate the relevance of the S100A14-CCL2/CXCL5 axis in clinical specimens, we examined the expression of S100A14, CCL2 and CXCL5 in our panel of 55 human patient samples. Consistently, tumors with high S100A14 expression levels exhibited robust staining for CCL2 and CXCL5 (**Figure 6A**). S100A14 expression was significantly correlated with CCL2 and CXCL5 expression (**Figure 6B**). Current studies demonstrated that S100A14 can be secreted into the extracellular space, which prompted us to evaluate whether S100A14 may be a potent serum biomarker in breast cancer. Thus, we detected the serum levels of S100A14, CCL2 and CXCL5 in our prospective cohort of 150 breast cancer patients and 125 healthy controls. The levels of these three proteins were higher in breast cancer patients than healthy controls (**Figure 6C**). Expectedly, serum S100A14 levels were positively correlated with CCL2 and CXCL5 levels, respectively (**Figure 6D**). Notably, the serum levels of these three proteins have significant diagnostic value for discerning breast cancer patients from healthy individuals. ROC curve analysis showed that the area under the curve (AUC) was 0.735, 0.754 and 0.697, the sensitivity was 71.3%, 71.3% and 65.3%, the specificity was 65.6%, 65.6% and 60.0% for S100A14, CCL2 and CXCL5, respectively (**Figure 6E**). We further examined whether the S100A14-CCL2/CXCL5 axis can serve as a predictor of the prognosis for patients with breast cancer.

Indeed, the GEO database analysis demonstrated that the increased expression of S100A14, CCL2 or CXCL5 in primary breast cancer was a determinant of poor metastasis-free survival in patients (**Figure S4**). Notably, the combined analysis of S100A14 and CCL2 or CXCL5 markedly discriminated the prognosis (**Figure 6F**). Collectively, these data demonstrate that S100A14 promotes breast cancer metastasis in an intracellular and extracellular manner. Mechanistically, S100A14 functions through increasing CCL2/CXCL5 expression via facilitating NF-κB translocation to the nucleus and activating the transcription of CCL2/CXCL5. Clinically, S100A14 drives breast cancer metastasis that is associated with an aggressive disease course and poor survival.

## Discussion

Tumor metastasis remains the main reason for breast cancer associated mortality 35. In spite of intensive research, the molecular driver events leading to tumor metastasis remain elusive. Identifying the key molecular events associated with tumor metastasis will allow for the development of new treatment solutions. Here, we provide compelling evidence that S100A14 acts as a prominent promoter of breast cancer cell and macrophage migration and invasion. Mechanistic investigations revealed that S100A14 facilitates the translocation of NF-κB from the cytoplasm to the nucleus, enhancing the binding of NF-κB to the promoters of CCL2/CXCL5 and upregulating their expression and secretion. Previous studies have shown that other S100 family members such as S100A7 and S100A9 regulate expression of chemokines and cytokines via different molecular mechanisms. While S100A7 triggers a cytokine network via a RAGE-mediated signaling cascade 36, S100A9 binds the EMMPRIN receptor and requires the adaptor protein TNF receptor-associated factor 2 (TRAF2) to upregulate the expression of TNFα, IL1 and IL6 etc 37. With regard to S100A14, we have previously reported that it binds to RAGE to activate MAPK and NF-κB signaling pathways. The CCL2-CCR2 axis has been reported to be involved in recruiting monocytes to the lung and promote breast cancer metastasis 30. In our study, we found S100A14 may promote macrophage M2 activation and recruitment through CCL2-CCR2 axis.

Evidence from *in vivo* models further validated that S100A14-CCL2/CXCL5 axis acts as a metastasis driver. In both orthotopic and knockout mouse models, S100A14 promotes metastasis but has no effect on tumor growth, suggesting that S100A14 specifically modulates tumor metastasis. Remarkably, we identified that the exosome-derived S100A14 plays a novel role in promoting cancer cell and macrophage migration and invasion and subsequent metastasis. Although previous literatures showed that several S100 proteins can be packaged into exosomes to activate PI3K/AKT and NF-κB signaling pathways 38, 39, we for the first time showed that S100A14 in exosomes can be transferred to cancer cells and macrophages and promotes breast cancer metastasis. However, how S100A14 enhances breast cancer metastasis via exosomal pathway still needs to be further studied.

The clinical relevance of the S100A14-CCL2/CXCL5 axis was examined by analysis of patient data from our own samples and public database. Our IHC analysis showed that S100A14 expression is elevated in breast cancer tissues compared with the matched adjacent normal tissues. It has been reported that the expression of S100 family members in human cancers is controlled by a complex regulatory network, which includes epigenetic mechanisms and signal transduction pathways. For example, epigenetic changes regulate S100P expression in prostate and pancreatic cancer 40, 41. S100A8 and S100A9 expression can be induced via RAGE activation in skin carcinogenesis 42. By analyzing the TCGA data, we found that the S100A14 gene is amplified in breast cancer, and the frequency of amplification is higher in metastatic tumors than the primary tumors. Moreover, the copy number amplification of S100A14 gene is significantly correlated with its expression. These results suggest that S100A14 overexpression could, at least in part, be ascribed to gene copy number amplification. Importantly, S100A14 expression is highly associated with CCL2 and CXCL5 expression in breast cancer tissue and serum samples, supporting our hypothesis that S100A14 regulates CCL2/CXCL5 expression in a physiological context. Notably, the serum levels of these three proteins have significant diagnostic value for discerning breast cancer patients from healthy individuals. Analysis of published datasets found that the coexpression of S100A14 and CCL2/CXCL5 in breast cancer tissues is a significant determinant of poor metastasis-free survival for breast cancer patients. Although we also collected our own serum specimens, it is difficult to evaluate the correlation between S100A14, CCL2 and CXCL5 serum levels and the prognosis of patients since the follow-up time is relatively short. In the future, our findings should be validated in larger, prospective cohorts to evaluate these three proteins as potential serum biomarkers of survival outcomes.

It has been well established that chemokines are involved in all stages of tumorigenesis and cancer progression, including malignant transformation, tumor growth, angiogenesis, immune responses, stromal cells recruitment and metastasis 43. With the deepening of the study of cytokines, chemokine anticancer therapeutic strategies have entered clinical evaluation currently 44. While some studies reported that chemokine targeting leads to improved outcomes in a range of preclinical models 45-47, a recent study found that CCL2-neutralizing antibodies causes enhanced metastatic burden 13. These results suggest that the approach to directly target chemokines needs to be scrutinized. In the present study, we identified S100A14 as a novel upstream regulator of CCL2, administration of CCL2 blocking antibody led to decreased breast cancer metastasis, providing an alternative strategy for targeting the CCL2 signaling pathway. Currently, inhibitors directly targeting S100B and S100A9 have been in clinical trials for melanoma and prostate cancer, respectively 48, 49. Future development of the S100A14 inhibitors will be needed to target the S100A14-CCL2/CXCL5 signaling axis in metastatic breast cancer.

In summary, our results identify a S100A14- NF-κB -CCL2/CXCL5 signaling axis in promoting breast cancer metastasis. These studies will significantly increase our understanding of breast cancer metastasis, which thus provided a promising strategy in the treatment of distant metastases of breast cancer.

## Supplementary Material

Supplementary figures and tables 1-3.

Supplementary table 4.

## Abbreviations

S100A14: S100 Calcium Binding Protein A14
CCL2: C-C Motif Chemokine Ligand 2
CXCL5: C-X-C Motif Chemokine Ligand 5
TME: Tumor microenvironment
TAM: Tumor associated macrophages
TCGA: The Cancer Genome Atlas
GEO: Gene Expression Omnibus
ELISA: Enzyme Linked Immunosorbent Assay
IHC: Immunohistochemistry
RAGE: the Receptor of Advanced Glycation End products
