# Supplementary material for: A S100A14-CCL2/CXCL5 signaling axis drives breast cancer metastasis
Source: Theranostics. 2020 Apr 27;10(13):5687–703. doi: 10.7150/thno.42087 (PMC7255008; doi:10.7150/thno.42087)
Supplement: Supplementary file 1 — Supplementary figures and tables 1-3. [file thnov10p5687s1.pdf]

Supplementary materials

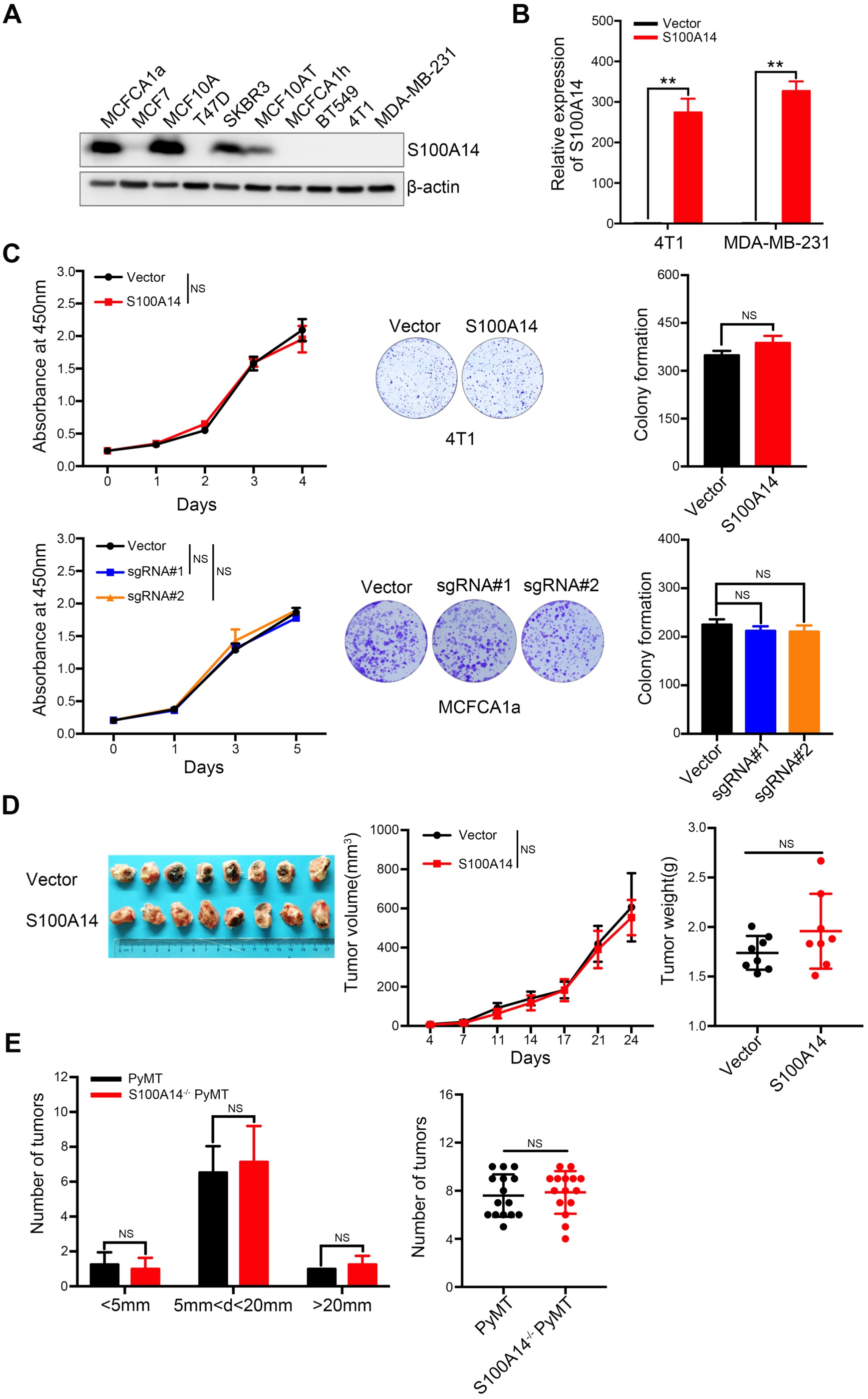

Figure S1. S100A14 has no effect on breast cancer cell growth *in vitro* and *in vivo*. (**A**) S100A14 expression was examined in nine breast cancer cell lines and one normal breast epithelial cell line by Western blot. β-actin was used as a loading control. (**B**) The efficiency of S100A14 overexpression was detected by qRT-PCR. (**C**) Left, cell proliferation was detected by CCK-8 assay after the ectopic overexpression of S100A14 in 4T1 cells (upper) and knockout of S100A14 in MCFCA1a cells (lower). Right, colony formation assays were performed after the ectopic overexpression of S100A14 in 4T1 cells (upper) and knockout of S100A14 in MCFCA1a cells (lower). Representative images and statistical analyses of number of colonies are shown. (**D**) The tumor pictures, tumor volumes and tumor weights of mice injected with S100A14-overexpressing 4T1 and control cells (n=8). (**E**) Statistical analyses of the number and diameter of tumors from $S100A14^{-/-}$ PyMT and PyMT mice. The birth date of PyMT mice was recorded. After 22 weeks, PyMT mice were killed, and the number and diameter of tumors were analyzed (n=15). Data in **C** and **D** are presented as the mean of biological replicates in a representative experiment ± SD. Data in **B** and **E** are presented as the mean ± SD, and *P* values are based on Student's *t*-test (2-sided). $^{**}P<0.01$. NS means no significant difference.

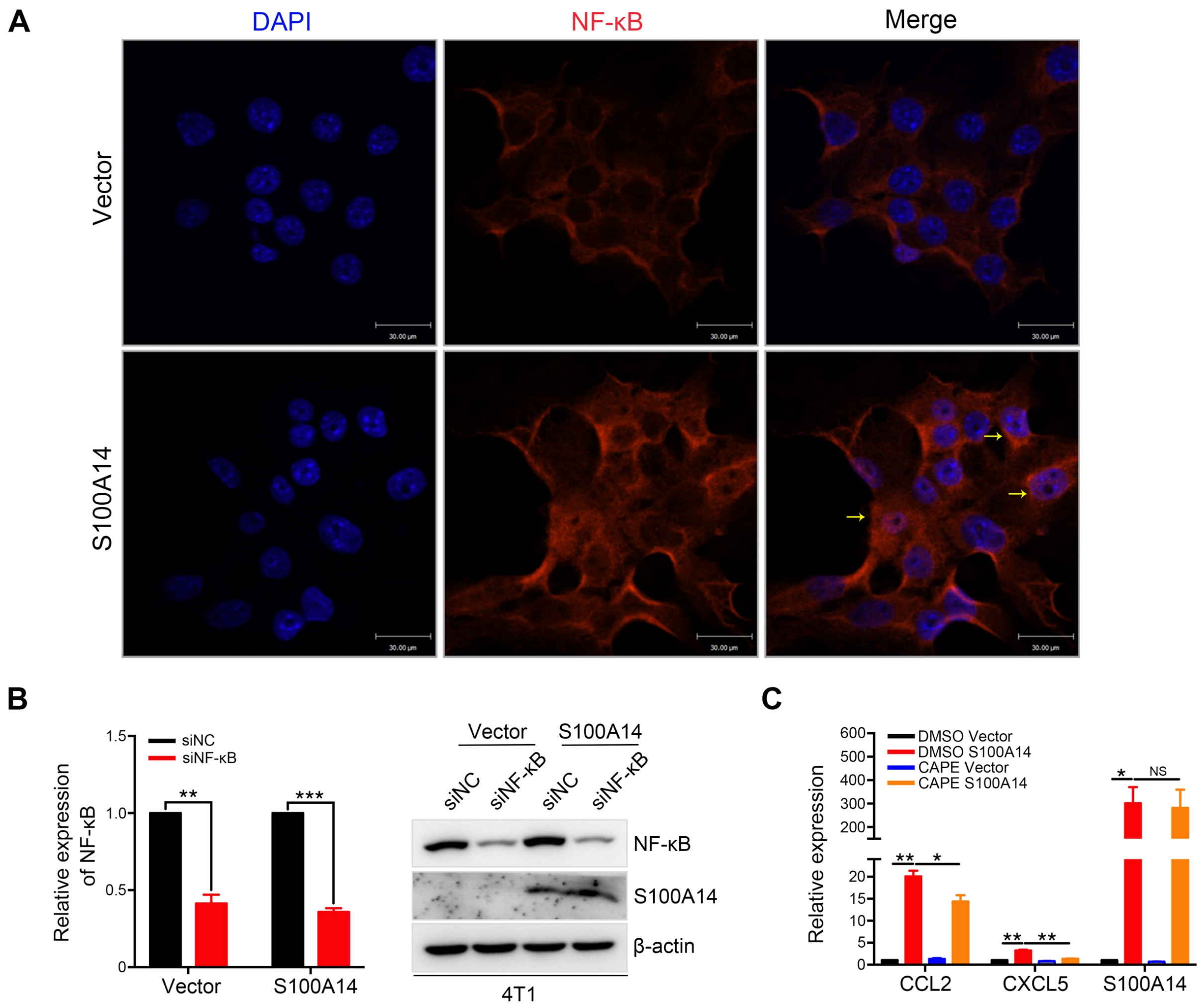

Figure S2. The expression of CCL2 and CXCL5 induced by S100A14 depends on NF-κB. **(A)** Immunofluorescence for detecting the distribution of NF-κB. Scale bars=30 μm. **(B)** S100A14-overexpressing 4T1 and control cells were transfected with siRNA (60 nM) for 24 h. The expression of NF-κB was detected by qRT-PCR (**Left**) and western blot (**Right**). (**C**) S100A14-overexpressing 4T1 and control cells were treated with CAPE (2 μM) and vehicle. After 48 h, the expression of S100A14, CCL2 and CXCL5 was detected by qRT-PCR. Data in **B** and **C** are presented as the mean ± SD; 2-sided t-test; *P<0.05, **P<0.01, ***P<0.001. NS means no significant difference.

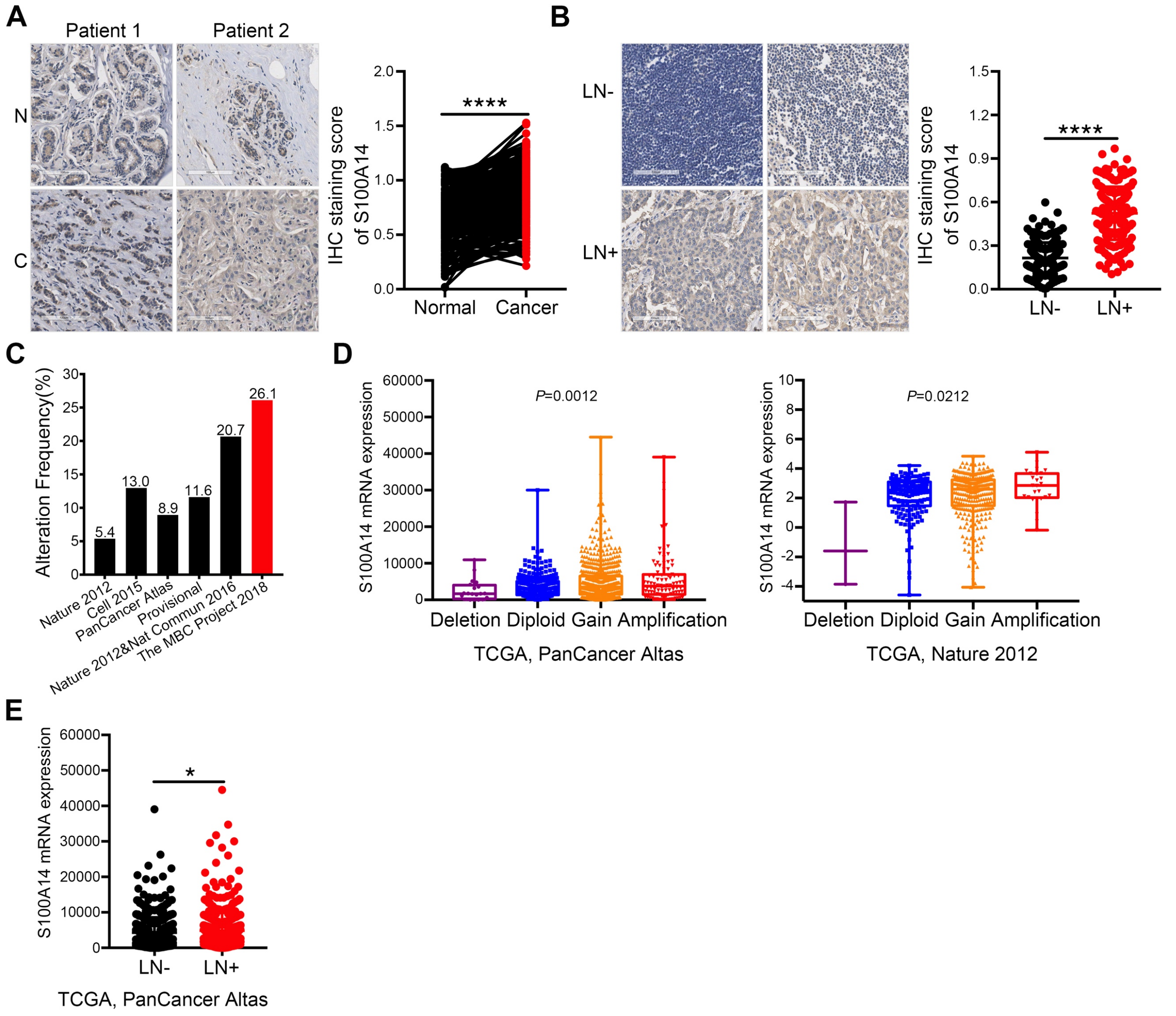

Figure S3. S100A14 displays copy number amplification and is overexpressed in breast cancer. (**A**) Histological analysis of S100A14 expression in the paired primary breast cancer tissues and adjacent normal tissues. Representative photographs (**left**) and statistical analyses (**right**) are shown. (**B**) Histological analysis of S100A14 expression in lymph node tissues with or without metastasis. Representative photographs (**left**) and statistical analyses (**right**) are shown. (**C**) The copy number amplification of S100A14 in breast cancer was analyzed using the TCGA dataset including METABRIC (Nature 2012), Breast Invasive Carcinoma (Cell 2015), PanCancer Atlas, Provisional, Nature 2012 & Nat Commun 2016 and The Metastatic Breast Cancer Project (Provisional, October 2018). From left to right, n = 778, 816, 2173, 1070, 1080, and 237. **(D)** The correlation between S100A14 amplification and its mRNA expression in the TCGA breast cancer dataset was analyzed

with the Kruskal-Wallis test. The mRNA expression values of S100A14 and the copy number variations were downloaded from the TCGA PanCancer Atlas and Nature 2012 dataset. **(E)** The correlation between S100A14 mRNA expression and lymph node metastasis was analyzed using the TCGA PanCancer Atlas dataset. Data in **A**, **B**, **D** and **E** are presented as the mean ± SD; paired or 2-sided t-test; **P*<0.05, *****P*<0.0001.

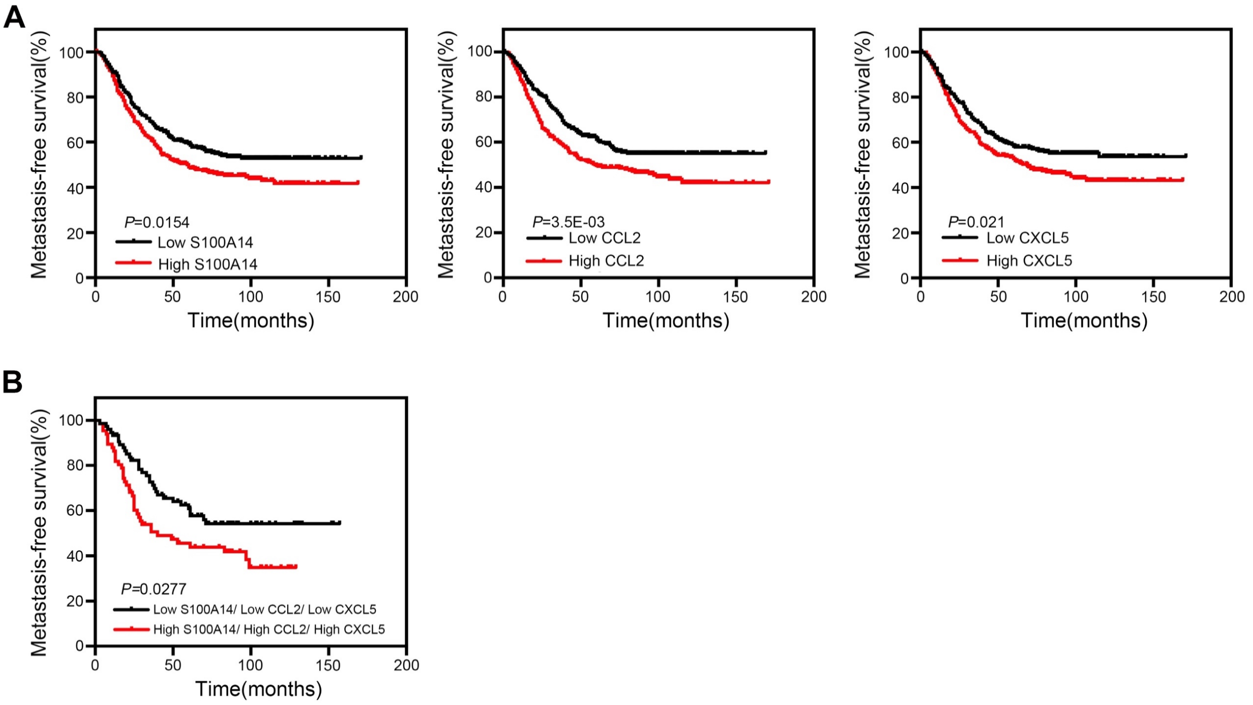

Figure S4. S100A14, CCL2 and CXCL5 expression can predict metastasis-free survival in breast cancer patients. Kaplan-Meier analysis for metastasis-free survival of breast cancer patients in the GEO database (GSE2034, GSE2603, GSE5327 and GSE12276). Patients were divided into two groups based on the median value for S100A14, CCL2 and CXCL5 (**A**). Patients were divided into two groups based on the median value for high S100A14 / high CCL2 / high CXCL5 and low S100A14/ low CCL2 / low CXCL5 (**B**). The log-rank test *P* values are shown.

Table S1. Primers, sgRNAs used in this study.

Name	Species	Forward Primer Sequence (5' to 3')	Reverse Primer Sequence (5' to 3')	Used for
S100A14	Human	TGCTCTAGAATGGGACAGTGTCGGTCAGCC	CGCGGATCCTCAGTGCCCCCGGACAGGCCT	Cloning Primer
S100A14	Mouse	GGAAGATCTAGTGTACCAGGAACGATGGGA	CCGCTCGAGTTCCAAGTAGAAGTCCTCAGC	Cloning Primer
S100A14 -sgRNA#1	Human	CACCGGGCTGACCGACACTGTCCCA	AAACTGGGACAGTGTCGGTCAGCCC	sgRNA
S100A14 -sgRNA#2	Human	CACCGTCCCACCCTCCACGGAGTAC	AAACGTACTCCGTGGAGGGTGGGAC	sgRNA
S100A14	Human	CTGACCCCTTCTGAGCTACG	TTCTCTTCCAGGCCACAGTT	RT-PCR Primer
β-actin	Human	AGGCACCAGGGCGTGAT	GCCCACATAGGAATCCTTCTGAC	RT-PCR Primer
S100A14	Mouse	ATGGGACAGTGTCGGTCAG	CCGCCACAGAGTATTTATGGAAG	RT-PCR Primer
β-actin	Mouse	CATTGCTGACAGGATGCAGAAGG	TGCTGGAAGGTGGACAGTGAGG	RT-PCR Primer
IL1A	Mouse	CGAAGACTACAGTTCTGCCATT	GACGTTTCAGAGGTTCTCAGAG	RT-PCR Primer
CXCL1	Mouse	TCCAGAGCTTGAAGGTGTTGCC	AACCAAGGGAGCTTCAGGGTCA	RT-PCR Primer
CXCL3	Mouse	CCATCCAGAGCTTGACGGTGAC	GGCTCAGCTGGACTTGCCGCTC	RT-PCR Primer
CXCL5	Mouse	TGCGTTGTGTTTGCTTAACCG	CTTCCACCGTAGGGCACTG	RT-PCR Primer
Cav1	Mouse	GCGACCCCAAGCATCTCAA	ATGCCGTCGAAACTGTGTGT	RT-PCR Primer
Rab25	Mouse	ATGGGGAATCGAACAGATGAAGA	GAGAACTCAACCCCGATGGT	RT-PCR Primer
Csf3	Mouse	ATCCCGAAGGCTTCCCTGAGTG	AGGAGACCTTGGTAGAGGCAGA	RT-PCR Primer
PSMB8	Mouse	GTGCAGGTTGTATTATCTTCGGA	CGAGTCCCATTGTCATCTACG	RT-PCR Primer
NTN1	Mouse	GGTCGGAGGAAACCTTGTTATC	ATTCCGTAGTTGTTGGATGCC	RT-PCR Primer
CCL2	Mouse	GTTGGC TCAGCCAGATGCA	AGCCTACTCATTGGGATCATCTTG	RT-PCR Primer
DAPK2	Mouse	CCCAGAAGGAGTCGTTAAGTGA	GGCTTGAGATCAAAGTGAGCAAT	RT-PCR Primer
Mtus1	Mouse	GCTGGGAAAACAAGCCACGAATG	AGTCTGGCTTGGAACTGCTCCT	RT-PCR Primer
MMP13	Mouse	CTTCTTCTTGTTGAGCTGGACTC	CTGTGGAGGTCACTGTAGACT	RT-PCR Primer
Hspb1	Mouse	GCTCACAGTGAAGACCAAGGAAG	TGAAGCACCGAGAGATGTAGCC	RT-PCR Primer

CCL2	Mouse	CCGATGGAGCTGCATGTATATCA	CCCTCTTTATTGGACCGAAGAGT	RT-PCR Primer for ChIP
CXCL5	Mouse	GCAAAGAGCCACTCCTCCTAGCC	ACCCACTGCACCCCTTTTATCTG	RT-PCR Primer for ChIP
Arg1	Mouse	CATTGGCTTGCGAGACGTAGAC	GCTGAAGGTCTCTTCCATCACC	RT-PCR Primer
iNOS	Mouse	GAGACAGGGAAGTCTGAAGCAC	CCAGCAGTAGTTGCTCCTCTTC	RT-PCR Primer
IL12	Mouse	CAATCACGCTACCTCCTCTTTT	CAGCAGTGCAGGAATAATGTTTC	RT-PCR Primer
CD206	Mouse	CTCTGTTCAGCTATTGGACGC	TGGCACTCCCAAACATAATTTGA	RT-PCR Primer
MRC1	Mouse	GTTCACCTGGAGTGATGGTTCTC	AGGACATGCCAGGGTCACCTTT	RT-PCR Primer
MRC2	Mouse	TACAGCTCCACGCTATGGATT	CACTCTCCCAGTTGAGGTACT	RT-PCR Primer
Fizz1	Mouse	CTTGTGGCTTTGCCTGTGGA	GCAGTGGTCCAGTCAACGAG	RT-PCR Primer
Ym1	Mouse	AGAAGGGAGTTTCAAACCTGGT	GTCTTGCTCATGTGTGTAAGTGA	RT-PCR Primer
MGL1	Mouse	TGAGAAAGGCTTTAAGAACTGGG	GACCACCTGTAGTGATGTGGG	RT-PCR Primer
MGL2	Mouse	TTAGCCAATGTGCTTAGCTGG	GGCCTCCAATTCTTGAAACCT	RT-PCR Primer
TNFα	Mouse	GGTGCCTATGTCTCAGCCTCTT	GCCATAGAACTGATGAGAGGGAG	RT-PCR Primer
TNFβ	Mouse	TGATACGCCTGAGTGGCTGTCT	CACAAGAGCAGTGAGCGCTGAA	RT-PCR Primer
IL6	Mouse	TACCACTTCACAAGTCGGAGGC	CTGCAAGTGCATCATCGTTGTTC	RT-PCR Primer
Loxp1	Mouse	CTTCTGTTATGAGGGTTTGCGCC	GAAGACACTCTAGAGGCCAGAATCCTG	Genotype identification
Loxp2	Mouse	TCTGTAACAGGTGATGCTGAA	CTAGACTTCTCCAACGAGGC	Genotype identification
CMV-Cre	Mouse	ATTTGCCTGCATTACCGGTC	ATCAACGTTTTCTTTTCGG	Genotype identification
KO#1	Mouse	CCTCATGTGGGTTGTATCTCCTTCAG	CCAAGTAGAAGTCCTCAGCTCCGAGT	Genotype identification
KO#2	Mouse	CTTCTGTTATGAGGGTTTGCGCC	CTAGACTTCTCCAACGAGGC	Genotype identification
MMTV	Mouse	GGAAGCAAGTACTTCACAAGGG	GGAAAGTCACTAGGAGCAGGG	Genotype identification
MMTV	Mouse	CAAATGTTGCTTGTCTGGTG	GTCAGTCGAGTGCACAGTTT	Genotype identification

Table S2. siRNAs used in this study.

Name	Species	Sequence (5' to 3')	Used for
ON-TARGETplus SMARTpool	Mouse	GCUCAAGAUCUGCCGAGUA	knock-down NF-κB
		GGCAUGCGAUUCCGCUAUA	
		GGGAUGAGAUCUUCUUGCU	
		CCAGACCGCAGUAUCCAUA	
ON-TARGETplus Control siRNA	Mouse	UGGUUUACAUGUCGACUAA	Negative control
		UGGUUUACAUGUUGUGUGA	
		UGGUUUACAUGUUUUCUGA	
		UGGUUUACAUGUUUUCCUA	

Table S3. Antibodies used in this study.

Antibody	Catalogue Number	Purpose
S100A14	NBP1-90000, Novus	WB IHC
NF-κB p65	8242, CST	ChIP IF WB
Hsp70	EXOAB-Hsp70A-1, SBI	WB
CD9	ab92726, Abcam	WB
CD81	sc-166029, Santa Cruz	WB
TSG101	14497-1-AP, Proteintech	IHC
Lamin B1	66095-1-Ig, Proteintech	WB
β-tubulin	66240-Ig, Proteintech	WB
β-actin	A5316, Sigma-Aldrich	WB
CCL2	GTX60582, GeneTex	IHC
CXCL5	AF254, Novus	WB
F4/80	GB11027, Servicebio	IHC
